# Genetic and Physical Mapping of DNA Replication Origins in Haloferax volcanii

**DOI:** 10.1371/journal.pgen.0030077

**Published:** 2007-05-18

**Authors:** Cédric Norais, Michelle Hawkins, Amber L Hartman, Jonathan A Eisen, Hannu Myllykallio, Thorsten Allers

**Affiliations:** 1 Institut de Génétique et Microbiologie, Université Paris-Sud, Orsay, France; 2 CNRS, UMR8621, Orsay, France; 3 Institute of Genetics, University of Nottingham, Nottingham, United Kingdom; 4 Johns Hopkins University, Baltimore, Maryland, United States of America; 5 The Institute for Genomic Research, Rockville, Maryland, United States of America; Hutchison/Medical Research Council Research Centre, United Kingdom

## Abstract

The halophilic archaeon Haloferax volcanii has a multireplicon genome, consisting of a main chromosome, three secondary chromosomes, and a plasmid. Genes for the initiator protein Cdc6/Orc1, which are commonly located adjacent to archaeal origins of DNA replication, are found on all replicons except plasmid pHV2. However, prediction of DNA replication origins in H. volcanii is complicated by the fact that this species has no less than 14 *cdc6/orc1* genes. We have used a combination of genetic, biochemical, and bioinformatic approaches to map DNA replication origins in *H. volcanii.* Five autonomously replicating sequences were found adjacent to *cdc6/orc1* genes and replication initiation point mapping was used to confirm that these sequences function as bidirectional DNA replication origins in vivo. Pulsed field gel analyses revealed that *cdc6/orc1*-associated replication origins are distributed not only on the main chromosome (2.9 Mb) but also on pHV1 (86 kb), pHV3 (442 kb), and pHV4 (690 kb) replicons. Gene inactivation studies indicate that linkage of the initiator gene to the origin is not required for replication initiation, and genetic tests with autonomously replicating plasmids suggest that the origin located on pHV1 and pHV4 may be dominant to the principal chromosomal origin. The replication origins we have identified appear to show a functional hierarchy or differential usage, which might reflect the different replication requirements of their respective chromosomes. We propose that duplication of H. volcanii replication origins was a prerequisite for the multireplicon structure of this genome, and that this might provide a means for chromosome-specific replication control under certain growth conditions. Our observations also suggest that H. volcanii is an ideal organism for studying how replication of four replicons is regulated in the context of the archaeal cell cycle.

## Author Summary

Haloferax volcanii is a member of the archaea, which are renowned for thriving in extreme environments. Archaea have circular chromosomes like bacteria but use enzymes similar to those found in eukaryotes to replicate their DNA. Few archaeal species have systems for genetics, and this has limited our understanding of DNA replication. We used genetics to map the chromosomal sites (origins) at which DNA replication initiates in *H. volcanii.* This species has a multipart genome comprising one main chromosome, three secondary chromosomes, and a plasmid. Five DNA replication origins were found and confirmed to function in vivo. All are adjacent to genes for the initiator protein Cdc6/Orc1, a common feature of archaeal replication origins. Two of the sequences are located on the main chromosome, confirming that multiple origins are often used to replicate circular chromosomes in archaea. Intriguingly, one of the origins from a secondary chromosome appears “dominant” to the principal chromosomal origin, suggesting either a hierarchy or differential usage of origins. This might reflect the different replication requirements of their respective chromosomes. Given the ease of genetic manipulation, H. volcanii holds great promise for studying how replication of four chromosomes is regulated in the context of the archaeal cell cycle.

## Introduction

In all prokaryotic organisms, and in certain unicellular eukaryotes, DNA replication is thought to initiate at well-defined chromosomal sites. These origins of replication serve as assembly sites for the protein machinery that unwinds the DNA duplex and initiates bidirectional DNA synthesis [1,2]. Bacterial chromosomes typically carry a single replication origin *(oriC)* and cognate initiator protein (e.g., DnaA), whereas eukaryotic chromosomes contain large numbers of replication origins, which are bound by a multiprotein origin recognition complex (ORC). Archaea use DNA replication proteins similar to those of eukaryotes but have circular chromosomes like bacteria [3]. Relatively little is known about origin utilization in archaea, and the available data suggest major differences in how chromosomes are replicated in the key archaeal groups. In particular, the chromosome of Pyrococcus abyssi (Euryarchaeota) is replicated from a single *oriC* [4], whereas three different *oriC*s are used to replicate the single chromosome of *Sulfolobus* species (Crenarchaeota) [5,6].

Archaeal replication origins consist of a long intergenic sequence containing an A/T-rich duplex unwinding element (DUE), which facilitates the local duplex opening required for replication fork assembly. The intergenic region is typically located upstream of a *cdc6/orc1* gene, which encodes a putative initiator protein that is homologous to both a subunit (Orc1) of eukaryotic ORC and the helicase loader Cdc6. Protein complexes formed by archaeal initiator proteins could therefore have a dual function in origin recognition and loading of minichromosome maintenance (MCM) helicase at the origin. However, available biochemical data are consistent with a role in origin recognition only. The intergenic region of the replication origin also carries multiple conserved sequence elements (origin recognition boxes, ORB) that are bound by Cdc6/Orc1 initiator proteins [6,7]. Binding of initiator proteins at the archaeal origin has been shown to proceed in a cooperative manner [8], suggesting that in archaea a defined multimeric initiator protein complex forms at the origin. Direct support for this idea has come from recent experiments indicating that higher-order assembly of Aeropyrum pernix Orc proteins results in structural and topological changes in the origin DNA [9].

Current knowledge of archaeal DNA replication is based on biochemical observations, and genetic studies are needed to test the extant models. In this respect, haloarchaea are ideal model organisms since they are easily cultured and amenable to genetic manipulation [10,11]. The genome sequences of Halobacterium sp. NRC-1 and Haloarcula marismortui have revealed that haloarchaea contain multiple replicons, each with essential genes [12,13]. However, the mechanisms that coordinate the replication of these multiple replicons remain unknown.

The genome of Haloferax volcanii has a multireplicon structure consisting of a main chromosome of 2.9 Mb and four smaller replicons (pHV1 [86 kb], pHV2 [6.4 kb], pHV3 [442 kb], and pHV4 [690 kb]) [14]. Like other haloarchaea, the H. volcanii genome encodes numerous putative *cdc6/orc1* genes that are distributed amongst the different replicons (except pHV2) (Table 1). This suggests that lineage-specific duplication of replication origins and/or initiator proteins has driven dynamic genome evolution in haloarchaea. However, previous experimental studies have identified only one likely replication origin on the main chromosome of the related haloarchaeon Halobacterium sp. NRC-1 [15].

**Table 1:**
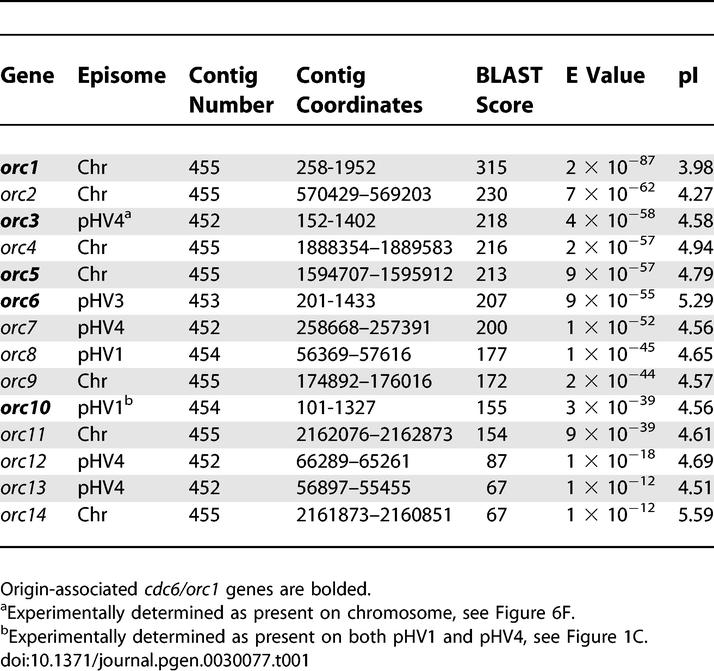
H. volcanii Cdc6/Orc1 Homologues: Result of BLAST Search with Cdc6/Orc1 Consensus COG1474.1 on H. volcanii Genome Sequence

**Figure 1:**
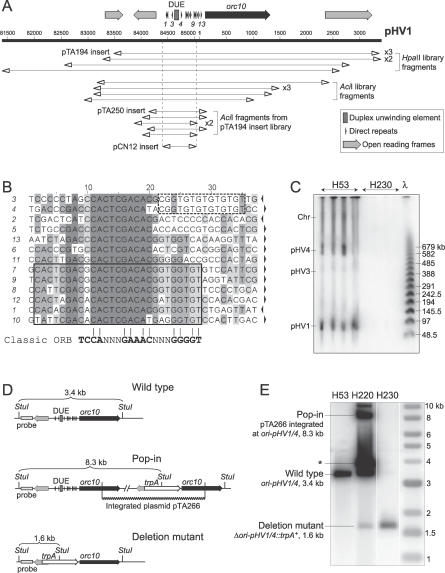
DNA Replication Origin on Contig 454 Is on Chromosomes pHV1 and pHV4 (A) Sequence features of the ARS element isolated from genomic libraries of WR340 DNA. Coordinates of plasmid inserts generated by HpaII and AciI digestion are shown (including insert in pTA194), in addition to the minimal ARS element determined by AciI digestion (in pTA250) or PCR amplification (in pCN12). Numbering refers to TIGR contig 454 (pHV1). (B) Sequence of repeats found at the intergenic region of the pHV1/4 replication origin (correspond to numbered arrows in Figure 1A). Orientation is indicated by arrows (righthand side) and conserved sequences are shaded. Six of the 13 repeats feature a complete ORB element (boxed), while a core mini-ORB element is conserved in all repeats. The sequence motif found in repeats surrounding the DUE is indicated by the dashed box. (C) Southern blot of PFG of intact DNA from strains H53 and H230, probed with HpaII ARS insert from pTA194, or intergenic region replaced by *trpA* in pTA266 (see Figure 1D). (D) Intergenic region of *ori-pHV1/4* was replaced by *trpA* marker by using the deletion construct pTA266. H. volcanii H53 was transformed with pTA266 to generate H220, which was used to derive the pHV1/4 origin deletion strain H230. Predicted fragment sizes of StuI digest are indicated. (E) StuI digest of genomic DNA from strains H53, H220, and H230, probed with DNA flanking the intergenic region. The band indicated * represents episomal DNA carrying the pHV1/4 origin, resulting from excision of the integrated plasmid.

Analysis of the genome sequence of H. volcanii (Hartman et al., unpublished data) suggested the presence of numerous replication origins, prompting us to search for autonomously replicating sequence (ARS) elements corresponding to each replicon. Replication initiation point (RIP) mapping was used to confirm that these ARS elements are functional in their chromosomal context. Our genetic data suggest a genome-wide hierarchy of some ARS elements, raising the possibility of chromosome-specific origin regulation in halophilic archaea. This study also provides a framework for investigating how the coordinated replication of four replicons is achieved in halophilic archaea.

## Results

Almost all origins of DNA replication identified in archaea to date are adjacent to genes coding for a homologue of eukaryotic Cdc6 and Orc1 proteins [16]. In order to identify putative replication origins in *H. volcanii,* we performed a TBLASTN search of the genome sequence of H. volcanii strain DS2 (http://www.tigr.org), using the consensus Cdc6/Orc1 sequence (COG1474.1) as a query. A total of 14 potential *cdc6/orc1* genes were found on the five replicons (Table 1), although one *(orc11)* is missing sequences coding for the C-terminal winged-helix domain that is required for DNA binding [17]. The large number of *cdc6/orc1* genes found in the H. volcanii genome parallels the situation in other halophilic archaea; ten *cdc6/orc1* genes are found in Halobacterium sp. NRC-1 (three replicons) and 17 are present in H. marismortui (nine replicons) [12,15]. Three of the ten putative origins in Halobacterium sp. NRC-1 were examined in a previous study, and only one (associated with *orc7*) proved capable of supporting autonomous replication of a plasmid [15]. Thus, it is unlikely that all the *cdc6/orc1* genes in H. volcanii are adjacent to active origins of DNA replication.

### Genetic Screen for DNA Replication Origins

To avoid any a priori bias regarding the location of replication origins, we carried out a genetic screen for ARS elements from *H. volcanii.* A partial HpaII digest was performed with genomic DNA from H. volcanii strain WR340 [18], which like all laboratory strains of H. volcanii lacks the smallest replicon pHV2 [19,20]. DNA fragments of between 4 and 8 kb were cloned in the nonreplicating *pyrE2*-marked plasmid pTA131 [21] and used to transform the *ΔpyrE2 ΔradA* strain H49 to prototrophy for uracil (*pyrE2+*). *H. volcanii radA* mutants such as H49 are defective in homologous recombination [22]; this precaution was taken to prevent integration of the plasmid into the genome by homologous recombination.

A total of 35 transformants were obtained. Plasmid DNA from seven transformants was sequenced, and in all cases, the insert corresponded to the same sequence on contig number 454 (Figure 1A). Since H. volcanii has more than one replicon, it was striking that only one ARS element was recovered. To ensure this was not a technical artifact of the screen, we repeated the library construction using AciI to generate the partial digest of H. volcanii genomic DNA and transformed H49 as before. Plasmid DNA from six AciI library transformants was sequenced, and all contained the same region of the H. volcanii genome isolated in the initial screen. This region comprises two divergently transcribed genes including *orc10,* separated by a 910-bp intergenic region featuring a 104-bp A/T-rich putative DUE (68% A/T versus 33% for overall genome) and several direct repeats (Figure 1A). Similar features are encountered at almost all characterized archaeal origins of DNA replication (e.g., [4]). However, it is notable that the nucleotide sequence of the direct repeats (Figure 1B) bears only little similarity to the ORB identified by Robinson et al. [6].

To determine the minimal sequence needed for DNA replication activity, we carried out a partial AciI digest of a representative 5-kb ARS insert that was cloned in plasmid pTA194 (Figure 1A). Fragments of 1–1.3 kb, 1.3–1.6 kb, 1.6–2 kb, 2–3 kb, and 3–4 kb were excised separately from an agarose gel and ligated with pTA131 to construct an ARS subclone library. Transformants of H. volcanii H49 were obtained with DNA from each size range. Plasmid DNA from six transformants in the 1–1.3-kb range was sequenced, and in all cases, the insert included the 910-bp intergenic region (Figure 1A). We were able to delimit the minimal origin further by amplifying a 633-bp fragment of the intergenic region using PCR (Figure 1A). This fragment was subcloned in pTA131 to generate pCN12 and used to transform the *ΔpyrE2 ΔradA* strain H112. Thus, the minimal origin is located in this region, and in contrast to what had been observed for Halobacterium sp. NRC-1 [15], the *cdc6/orc1* gene is not required in *cis* for ARS activity in *H. volcanii.*

We used the ARS insert in pTA194 to probe a Southern blot of intact H. volcanii DNA displayed on a pulsed field gel (PFG). Two bands were observed (Figure 1C), which correspond in size to replicons pHV4 (690 kb) and pHV1 (86 kb) [14]. The intensity of the two bands was similar, suggesting that this replication origin is present on both pHV1 and pHV4 and that both replicons are present in all cells. We employed the minimal ARS element from the AciI subclone library (in pTA250) to generate low copy–number shuttle vectors with *pyrE2, trpA, hdrB,* and *leuB* selectable markers [21] (Figure S1). The principal advantage of these shuttle vectors over existing plasmids based on the origin of replication from pHV2 is their smaller size (∼4.5 kb for pHV1/4-based vectors versus ≥7.5 kb for pHV2-based vectors).

### Deletion of Replication Origin from pHV1 and pHV4

The fact that only one ARS element was recovered in the initial genetic screen suggests that this sequence might be “dominant” and thereby prevent the isolation of other origins. We hypothesized that deleting this sequence from pHV1 and pHV4 by a gene knockout system [18,21] would allow the isolation of ARS elements corresponding to origins of DNA replication on the main chromosome and pHV3. The pHV1/4 replication origin and adjacent genes were subcloned to generate pTA252, and a 1-kb fragment containing the intergenic region necessary for ARS activity was replaced by a *trpA* selectable marker for tryptophan biosynthesis [21]. This plasmid (pTA266, Figure 1D) was used to transform the *ΔpyrE2 ΔtrpA* strain H53, and integration at the *orc10* locus was verified by Southern blot (Figure 1E, strain H220). Counter-selection with 5-fluoroorotic acid (5-FOA) was used to ensure loss of integrated pTA266 by intramolecular recombination and to yield a *trpA*-marked deletion of the intergenic region (Figure 1D). While origin deletion events (*trpA^+^* 5-FOA-resistant cells) were obtained, they were outnumbered >1,000-fold by events leading to restoration of the wild-type (*trpA^–^* 5-FOA-resistant cells), indicating a strong bias for maintenance of the replication origin. The deletion was verified by Southern blots of a genomic DNA digest (Figure 1E, strain H230) and intact DNA displayed on a PFG (Figure 1C). The pHV1 and pHV4 replicons are still present (unpublished data) and must therefore be using alternative replication origins. Since the deletion strain H230 did not show any obvious growth defects, these alternative origins presumably act as sites of efficient DNA replication initiation. This observation prompted us to search for other ARS elements.

### Isolation of a Chromosomal Origin of DNA Replication

**Figure 2:**
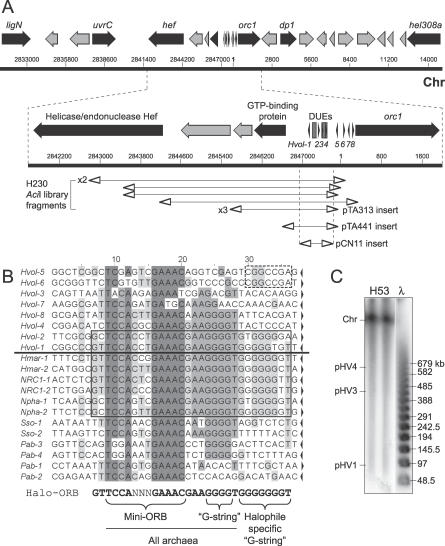
DNA Replication Origin on the Main Chromosome: *oriC1* (A) Sequence features of the ARS element isolated from genomic libraries of H230 DNA, including selected genes (see text for details). Coordinates of plasmid inserts generated by AciI digestion are shown (including insert in pTA313), in addition to the minimal ARS element in pTA441 and pCN11. See Figure 1A for key. Numbering refers to TIGR contig number 455. Main chromosome, Chr. (B) Above the line are sequences of repeats found at the intergenic region of the H. volcanii chromosomal replication origin (correspond to numbered arrows in Figure 2A). Below the line are sequences of repeats found at other (presumed) archaeal origins. The species and relevant *cdc6/orc1* genes are H. marismortui cdc6–4 (Hmar-1–2), Halobacterium sp. NRC-1 *orc7 (NRC1-1-2), Natronomonas pharaonis cdc6–1 (Npha-1–2), S. solfataricus cdc6–1 (Sso-1–2),* and *P. abyssi cdc6 (Pab-1–4).* The orientation is indicated by arrows and conserved positions are shaded. Among halophilic archaea, repeats surrounding the primary DUE feature a longer consensus sequence (Halo-ORB, boxed), which contains the core mini-ORB and “G-string” elements also found in other archaea, plus a halophile-specific “G-string.” (C) Southern blot of PFG of DNA from strain H53, probed with the AciI ARS insert from pTA313.

We repeated the screen outlined above using the deletion strain H230 as a source of genomic DNA. AciI was used for a partial digest of H230 genomic DNA, fragments of 3–5 kb were cloned in pTA131, and H49 was transformed as before. Plasmid DNA from nine transformants was sequenced, and in all cases the insert localized to a single region of the H. volcanii genome (contig number 455, which corresponds to the main chromosome). This region (Figure 2A) comprises two divergently transcribed genes including *orc1,* separated by a 1,360-bp intergenic region featuring two A/T-rich DUEs and several direct repeats. The nucleotide sequence of the direct repeats (Figure 2B) is 89% identical to the ORB sequence (euryarchaeal consensus 5′-GTTCCAGTGGAAAC-AAA‐‐‐‐GGGGG-3′) [6]. In addition to *orc1,* a number of other genes related to DNA replication and repair is found in the vicinity of the ARS (Figure 2A), encoding the DP1 exonuclease subunit of the archaeal D family DNA polymerase [23], the Hef helicase/endonuclease [24], a homologue of the bacterial UvrC nucleotide excision repair protein, an NAD-dependent DNA ligase [25], and the Hel308 helicase [26].

The ARS insert in one plasmid (pTA313) was used to probe a Southern blot of intact H. volcanii DN5A on a PFG. As expected, one band was observed (Figure 2C) that corresponds in size to the main chromosome (2.9 Mb) [14]. A 1.1-kb Sau3AI-HindIII fragment of pTA313, comprising the intergenic region only (Figure 2A), was subcloned in pTA131 to generate pTA441 and used to transform H49. Transformants were obtained with high efficiency indicating that, as with the pHV1/4 origin, the *cdc6/orc1* gene is not required in *cis* for ARS activity. To delimit the minimal origin further, a 692-bp fragment of the intergenic region was amplified by PCR (Figure 2A), subcloned in pTA131 to generate pCN11, and used to transform the *ΔpyrE2 ΔradA* strain H112. The sequence was able to maintain the plasmid.

### ARS Plasmids with pHV1/4 Replication Origin Are Dominant to, but Less Stable Than, Plasmids with Chromosomal Origin

**Figure 3:**
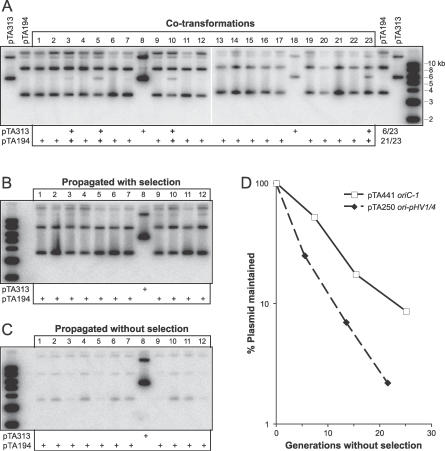
*ori*-*pHV1/4* ARS Plasmids Are Dominant to, but Less Stable Than, *oriC-1* ARS Plasmids (A) H112 was transformed with a mixture of 0.5 μg each of pTA194 and pTA313. Crude DNA was isolated from 23 transformants and a Southern blot probed with *pyrE2* sequences. (B) Transformants 1–12 were propagated by restreaking on selective Hv-Ca agar. DNA was isolated and probed as above. (C) Transformants 1–12 were restreaked on nonselective Hv-YPC agar. DNA was isolated and probed as above. (D) H112 containing pTA250 or pTA441 was grown in nonselective Hv-YPC broth. At regular intervals aliquots were plated on selective (Hv-Ca) and nonselective (Hv-YPC) agar, to determine the fraction of uracil+ cells.

To explain why only one ARS element was recovered in the initial genetic screen, we determined whether the replication origin located on pHV1 and pHV4 might be “dominant” to other origins. The *ΔpyrE2 ΔradA* strain H112 was transformed with an equimolar mixture of pTA194 and pTA313 (0.5 μg each). These are ARS plasmids from the initial and secondary genetic screens, which carry the pHV1/4 and chromosomal origins, respectively. A total of 23 transformants were analyzed by Southern blotting for the presence of each ARS plasmid (Figure 3). The vast majority (21/23) contained the *ori-pHV1/4* plasmid pTA194 and in most cases (17/23) this was the sole plasmid detectable. Only six transformants contained the *oriC* plasmid pTA313, and in most cases (4/6) pTA194 was also present. To determine the fate of ARS plasmids in transformants containing both pTA194 and pTA313, cells were propagated by restreaking, both while maintaining selection for the *pyrE2* marker and without selection. In all three cases analyzed (Figure 3B, transformants 3, 5, 10), only pTA194 remained after further propagation. This was not due to greater stability of pTA194 relative to pTA313, since transformants propagated without selection showed a marked loss of pTA194, whereas pTA313 was well maintained in the transformant where it was present exclusively (Figure 3C, transformant 8). These results recapitulate the outcome of the genetic screens and indicate that the *ori-pHV1/4* plasmid pTA194 is dominant to the *oriC* plasmid pTA313.

The stability of ARS plasmids in the absence of selection was investigated in a quantitative manner. Cultures of H112 transformed with pTA250 or pTA441, which carry the intergenic region of *ori-pHV1/4* and *oriC,* respectively, were propagated for ∼25 generations in nonselective Hv-YPC broth. At regular intervals, the fraction of uracil+ cells (indicative of ARS plasmids) was determined by plating on selective and nonselective media. As shown in Figure 3D, the *ori-pHV1/4* plasmid pTA250 is significantly less stable than the *oriC* plasmid pTA441; plasmid loss was calculated to be 16% per generation for pTA250 and 9% per generation for pTA441.

### GC-Skew Analyses Predict Additional Replication Origins

**Figure 4:**
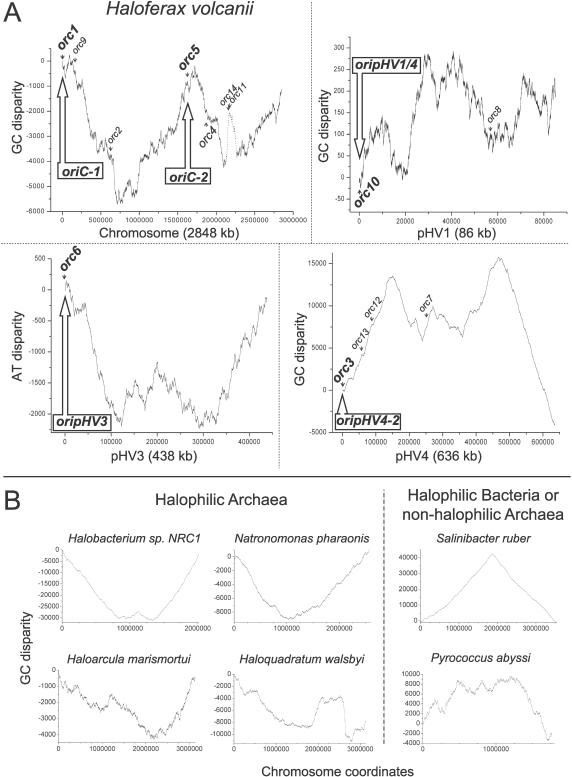
Nucleotide Disparity Curves (A) Nucleotide disparity curves of *H. volcanii* DS2 genome sequence*.* Positions of the 14 *cdc6*/*orc1* genes and five replication origins identified in this work are indicated. A putative prophage sequence with high A + T content results in a sharp peak of nucleotide disparity for the main chromosome (indicated by dotted line), but does not coincide with any ARS element isolated here. Nucleotide skews are calculated using the ZPLOTTER program (http://tubic.tju.edu.cn/zcurve). (B) GC disparity curves of the chromosome of four halophilic archaea, a halophilic bacterium, and a non-halophilic archaeon. Chromosome coordinates have been offset to begin at H. sp. NRC-1 *orc7, N. pharaonis cdc6, H. marismortui cdc6–4, H. walsbyi cdc6–1, S. ruber dnaA,* and *P. abyssi cdc6* genes, which are assumed or proven to be associated with the replication origins. The halophilic bacterium and non-halophilic archaeon were used as a control.

The leading and lagging strands of bacterial and archaeal genomes differ in their base composition [27,28], and a surplus of G over C is usually found on the leading strands of replication. Thus, abrupt changes in the strand-specific nucleotide compositions indicate the presence of a replication origin or terminus. We analyzed the G/C disparity of the largest contig (number 455) of the H. volcanii genome sequence, which corresponds in size to the main chromosome (2.9 Mbp). Two different algorithms, ORIGINX [29] (unpublished data) and Z-CURVE (Figure 4A) [30] gave similar results. The “GC-skew” of H. volcanii suggests the presence of multiple origins on the main chromosome, similar to what has been proposed for Halobacterium sp. NRC-1 [30,31]. The amplitude of the calculated H. volcanii GC-skew was similar to that of *H. marismortui,* but substantially smaller than was observed for the normalized GC-skews of the main chromosomes of three other halophilic archaea and a non-halophilic archaeon (Figure 4B). Whereas the *oriC* of the halophilic bacterium Salinibacter ruber has a well-defined GC-skew minimum at the origin, the four halophilic archaea show a GC-skew with inverted polarity, when compared to bacteria and thermophilic archaea. Therefore, the leading strand of replication contains an excess of C in haloarchaea, similar to what has been suggested for *Mycoplasma* species [32]. While the mechanisms that establish these skews at a molecular level are still poorly understood, it is of note that their formation seems to be independent of GC% of genomes [32]. For example, the chromosome of the haloarchaeon *Haloquadratum walsbyi,* which is not GC-rich (48% GC), also shows an inverted polarity.

The H. volcanii G/C disparity curve has three peaks for the main chromosome (Figure 4A): (1) A peak near the ARS element identified in genomic libraries from H230, which is associated with the *orc1* gene *(oriC1);* (2) A peak associated with the *orc11* and *orc14* genes. This chromosomal region has a base composition different than the rest of the genome and contains putative viral genes such as HNHc endonuclease, VirB4, and tragVirD4 helicases or bacteriophage T4-like integrase (unpublished data). Moreover, it does not coincide with any ARS elements isolated in this work; and (3) A peak associated with the *orc5* gene, suggesting the presence of a second replication origin on the main chromosome *(oriC2).* GC, AT, and G + C content disparities were also carried out for contigs corresponding to the smaller replicons and predicted the location of DNA replication origins for pHV1 (confirming the origin identified in the first ARS screen), pHV3, and a second origin on pHV4 (Figure 4A); all of these peaks are located near *cdc6/orc1* genes. Strikingly, *ori-pHV1/4* sequence could only be found on contig number 454 (pHV1) and not on contig number 452 (which should correspond in size to pHV4).

**Figure 5:**
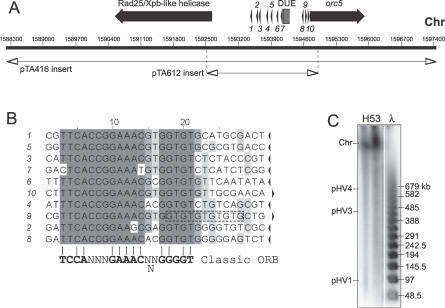
A Second DNA Replication Origin on the Main Chromosome: *oriC2* (A) Sequence features of the second origin from main chromosome. Coordinates of the 9.3-kb NotI genomic fragment cloned in pTA416 and the 2.4-kb intergenic region cloned in pTA612 are shown. See Figure 1A for key. Numbering refers to TIGR contig number 455. Main chromosome, Chr. (B) Sequence of repeats found at the intergenic region of *oriC2* (numbered arrows in Figure 5A). The orientation is indicated by arrows and conserved sequences are shaded. (C) Southern blot of PFG of DNA from strain H53, probed with intergenic region cloned in pTA612.

To test the possibility of a second chromosomal origin and to identify origins on the smaller replicons, we directly cloned candidate ARS elements. A region carrying the putative second chromosomal replication origin *(oriC2)* was isolated as a 9.3-kb NotI fragment from a genomic DNA library and was cloned in pTA416 (Figure 5A). This fragment contains two divergently transcribed genes coding for Orc5 and a putative Rad25/Xpb-related helicase, separated by a 2,094-bp intergenic region featuring an A/T-rich DUE and several direct repeats with similarity to the ORB consensus (Figure 5B). A 2.4-kb fragment comprising this intergenic region was amplified by PCR, cloned in pTA131 to generate pTA612, and used to transform the *ΔpyrE2 ΔradA* strain H112. Transformants were obtained with high frequency, indicating that this region supports ARS activity (Figure S2). However, after 15 d of incubation the colonies were significantly smaller than those seen with H112 transformed with pTA250 (pHV1/4 origin) or pTA441 *(oriC1).* This suggests that the efficiency of this putative second chromosomal origin (or the stability of the plasmid) is significantly lower than that of the pHV1/4 and *orc1*-associated origins. Moreover, the intergenic sequence upstream of *orc5,* which contains *oriC2,* can be deleted efficiently by using the same gene knockout procedure as was used to delete the pHV1/4 origin [21]. The resulting strain CN28 shows no growth defect (unpublished data).

**Figure 6:**
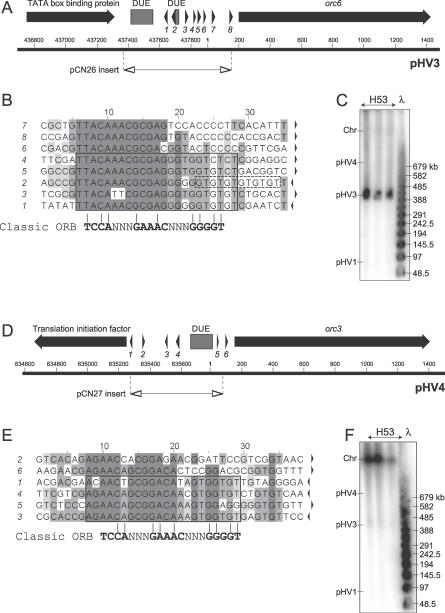
Additional ARS Elements Predicted by Nucleotide Composition (A) Sequence features of replication origin on pHV3. Coordinates of 693-bp intergenic region cloned in pCN26 are shown. See Figure 1A for key. Numbering refers to TIGR contig number 453 (pHV3). (B) Sequence of repeats found at the intergenic region of the pHV3 origin (numbered arrows in Figure 6A). The orientation is indicated by arrows and conserved sequences are shaded. (C) Southern blot of PFG of DNA from strain H53, probed with intergenic region cloned in pCN26. (D) Sequence features of ARS element in contig number 452. Coordinates of 592-bp intergenic region cloned in pCN27 are shown. Numbering refers to TIGR contig number 452 (pHV4). (E) Sequence of repeats found at intergenic region of contig number 452 ARS element (numbered arrows in Figure 6D). (F) Southern blot of PFG of DNA from strain H53, probed with intergenic region cloned in pCN27.

The 793-bp intergenic sequence on contig number 453 (corresponding to pHV3) is located between genes coding for a putative TATA box-binding protein and Orc6, and features two DUEs and several direct repeats with similarity to ORB consensus sequence (Figure 6A and 6B). To test if this intergenic region confers ARS activity, a 693-bp PCR fragment including the DUEs and repeats was inserted in pTA131 to generate pCN26 and used to transform H112. Transformants were obtained with high frequency (Figure S2). When probed with the *ori-pHV3* sequence, a PFG confirmed that this origin is located on the pHV3 replicon (Figure 6C).

The 688-bp intergenic region on contig number 452 (which should correspond to pHV4) is located between a putative translation initiation factor and *orc3* genes and features a DUE and multiple direct repeats that are different from the ORB consensus sequence (Figure 6D and 6E). This putative origin was tested for ARS activity by cloning a 592-bp PCR fragment of the intergenic sequence in pTA131 to generate pCN27 and used to transform H112. Significantly fewer transformants were obtained with pCN27 *(ori-pHV4–2)* than with pCN26 *(ori-pHV3)* or pTA250 *(ori-pHV1/4),* indicating that the second origin of pHV4 is not used efficiently (Figure S2). Moreover, a PFG probed with *ori-pHV4–2* sequence showed that this origin is actually located on the 2.9-Mb main chromosome (Figure 6F), suggesting that the strains used in our experiments and/or sequenced by TIGR may have undergone genome rearrangements.

The stability of ARS plasmids pTA612 *(oriC2),* pCN26 *(ori-pHV3),* and pCN27 *(ori-pHV4–2)* was determined in a qualitative manner. Six transformants of H112 containing each ARS plasmid were grown on nonselective agar and then restreaked on selective medium. After restreaking, none of the transformants showed growth on selective agar, indicating complete loss of the plasmid (Table 2). In comparison, transformants with pTA441 *(oriC1)* showed normal growth on selective agar, indicating efficient maintenance of the plasmid. Transformants with pTA250 *(ori-pHV1/4)* showed growth on selective agar, but the number of colonies was significantly reduced relative to growth on nonselective agar, indicating some loss of the plasmid.

**Table 2:**
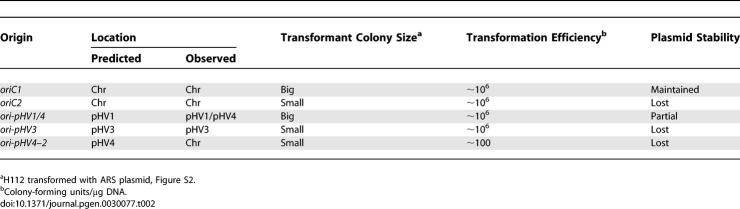
Characteristics of Replication Origins

As a control, we tested whether the intergenic region adjacent to *orc4* could function as an ARS element; this region does not coincide with a peak of G/C disparity (Figure 4A) and does not feature a DUE. A 1.7-kb ApaI-BglII genomic DNA fragment of this intergenic region was cloned in pTA131 to generate pTA611 and used to transform H112. No transformants were observed, indicating that not every *cdc6/orc1* gene is associated with a potential DNA replication origin.

### ARS Elements Function as Replication Origins

**Figure 7:**
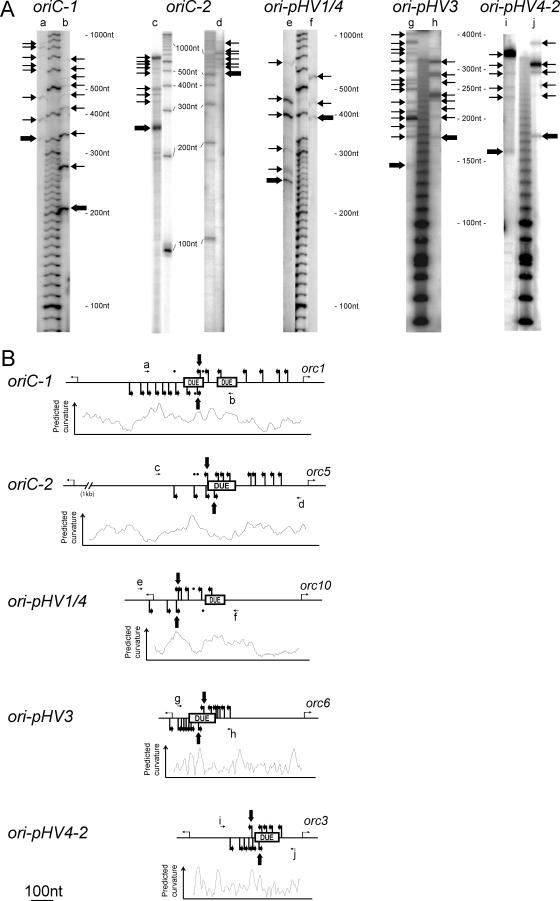
Mapping of Initiation Sites at the Replication Origins (A) Primer extension reactions were performed with enriched replicating intermediate DNA fragments and separated on a denaturing polyacrylamide gel. Corresponding primers are indicated on top of the gels (see Table S1 for details), arrows refer to the 5′ end of amplification products and transition points are indicated by bold arrows. (B) Primers and detected initiation sites along the sequence. The positions of DUEs, the associated *cdc6/orc1* gene on the right, and the upstream open reading frame on the left are shown for each replication origin. Dots indicate weak amplification products obtained with control templates (linearized plasmid DNA carrying the replication origins, isolated from E. coli cells).

RIP mapping [33] was used to determine the exact positions of DNA replication initiation at the origins we had identified. A preparation of enriched nascent DNA strands was used in primer extension reactions using ^32^P-labeled primers hybridizing to the leading strand either side of the DUEs of each ARS element (Table S1). The sizes of the amplification products were determined using electrophoresis under denaturing conditions (Figure 7A), allowing identification of the shortest amplification products that mark the start site of the leading strand. Clear transition points between leading and lagging strand replication within or near the DUEs were revealed for both strands of each origin (Figure 7B). It is of note that the detected transition points colocalize with chromosomal regions where DNA is predicted to be bent. Amplification products obtained in negative control experiments, which used linearized plasmids containing the origin sequences that were isolated from *Escherichia coli,* are indicated in Figure 7B using dots. As expected, they do not coincide with the extension products obtained using H. volcanii replication intermediates. These results indicate that all the ARS elements isolated during this work are functional in their chromosomal context.

## Discussion

Initiation of DNA replication at an origin depends on *cis*-acting sequence elements. In bacteria and unicellular eukaryotes, these have been well-characterized using genetics. In contrast, archaeal replication origins were identified relatively recently [2], and only limited genetic methods have been used to study DNA replication in archaea [15]. In this work, we isolated several ARS elements that use chromosomally encoded factors for their replication. RIP assays were used to confirm that these ARS elements correspond to functional replication origins in their chromosomal context. The origins are distributed on the different replicons of *H. volcanii,* including two on the main chromosome. Previous work in Sulfolobus solfataricus and recent studies in A. pernix have shown that Crenarchaeota can use more than one origin to replicate a circular chromosome [5,6]. Bioinformatics has suggested that Halobacterium sp. NRC-1, a euryarchaeon like *H. volcanii,* might have two chromosomal replication origins [34], but attempts to identify the second origin experimentally were not successful [15]. Our work provides the first proven example from the Euryarchaeota of multiple origins per replicon.

The general characteristics of the H. volcanii replication origins are similar. All contain AT-rich sequences required for unwinding and/or bending of the origin DNA. These are surrounded by repeated sequence motifs that correspond to the classic ORB elements found at other archaeal origins. However, some of these repeats show significant differences to the archaeal mini-ORB consensus. Many archaeal ORBs also feature a characteristic “G-string” element (Figure 2) that contributes to Cdc6/Orc1 binding at the origin (S. Bell, personal communication). While “G-string” elements are found at all origins of *H. volcanii,* the two ORBs surrounding the primary DUE of *oriC1* also contain an extended “G-string” that appears to be specific to halophiles (Figure 2). Furthermore, long GT-stretches are found after some repeats, such as the two ORBs surrounding the DUE of *ori-pHV1/4* (Figure 1B). The subtle variations found in halophilic ORB sequences might contribute to origin-specific binding by different Cdc6/Orc1 proteins. Alternatively, these halophile-specific features could promote DNA bending at high intracellular salt concentrations, thus favoring the formation of a higher order complex between the origin and initiator proteins [9].

The number of Cdc6/Orc1 proteins is almost certainly higher than the number of origins in *H. volcanii;* for example, the intergenic region adjacent to *orc4* does not show ARS activity. Furthermore, we were able to delete several *cdc6/orc1* genes (*orc1, orc5,* and *orc10,* unpublished data), suggesting that their functions at least partially overlap. It is possible that some Cdc6/Orc1 proteins might promote the initiation of “routine” DNA replication, while other initiators are adapted to function under specific physiological conditions. For example, our observation that the second chromosomal origin *(oriC2)* appears to function less efficiently in laboratory conditions suggests that it is only used under certain circumstances. This could be due to location of the origins on ARS plasmids, as opposed to their native chromosomal loci. On the other hand, it is noteworthy that the overall pI values of the different Cdc6/Orc1 proteins vary between 3.98 and 5.59 (Table 1), with the *oriC1-*associated Orc1 being the most acidic, raising the possibility that they might function optimally under different salt concentrations.

A surprising result that emerged from our initial genetic screen for ARS elements was that only one origin *(ori-pHV1/4)* was isolated. This is unlikely to be due to technical limitations of the method, since extensive libraries were constructed using different restriction enzymes. Moreover, we were able to recapitulate the outcome of the screen by cotransforming H. volcanii with a mixture of *ori-pHV1/4* and *oriC1* ARS plasmids (Figure 3). The majority (74%) of transformants contained only the *ori-pHV1/4* plasmid, and less than 9% contained only the *oriC1* plasmid (Figure 3A). This disparity is unlikely to be due to differences in plasmid establishment, since the two ARS constructs show the same transformation efficiency (Table 2) and confer a similar phenotypic load on their host (Figure S2). Instead, it would appear that the *ori-pHV1/4* ARS plasmid is dominant to the *oriC1* ARS plasmid. Evidence in favor of this suggestion emerged from the examination of transformants containing both plasmids (Figure 3B). Upon further propagation, only the *ori-pHV1/4* ARS plasmid remained while the *oriC1* ARS plasmid was lost. This result is all the more intriguing given that *ori-pHV1/4* plasmids are significantly less stable than *oriC1* plasmids (Figure 3C and 3D). Furthermore, the copy number of *ori-pHV1/4* plasmids is lower than that of *oriC1* plasmids (unpublished data). Thus, our results cannot be explained by mere incompatibility of ARS plasmids, since this would favor the retention of the *oriC1* plasmid.

Instead, we suggest that in H. volcanii there is a functional hierarchy of replication origins, which arises from competition for common replication factors. “Dominant” origins such as *ori-pHV1/4* might be bound more efficiently by Cdc6/Orc1 or other proteins such as MCM helicase and primase that participate in replication initiation. Other factors might also contribute to differential usage of origins. ARS elements that initiate efficiently can be selectively enriched from a genomic library of Saccharomyces cerevisiae [35], and reduced initiation efficiency was found to correlate with transcriptional activity directed towards the ARS element. This suggests that transcriptional interference with the prereplication complex is a determinant of origin hierarchy in eukaryotes. It is noteworthy that four of the five origins identified in this work (and almost all other archaeal origins) feature transcription units that are orientated away from replication initiation sites (Figures 1, 2, 5, and 6). Therefore, transcriptional interference might also regulate origin firing in archaea.

Our genetic observations indicate that while the functions of initiator proteins at least partially overlap in *H. volcanii,* the replication origins *oriC1* and *ori-pHV1/4* play a key role in the replication of their corresponding chromosomes. This suggests that duplication of replication origins, and not of the initiator genes, could have allowed the H. volcanii genome to develop toward a multireplicon structure. This process might still be continuing, since there are important differences between our data and the assembled genome sequence. For instance, both pHV1 and pHV4 carry a similar (if not identical) replication origin. A comparable situation is found with Halobacterium sp. NRC-1 plasmids pNRC100 and pNRC200, which share a large region of around 150 kb including long (33 and 39 kb) inverted repeats [13]. An additional example is *ori-pHV4–2,* which in the current version of the assembled genome sequence is located on the contig corresponding to pHV4, while in our laboratory strains is carried on the main chromosome. Detailed manual examination of the genome sequencing data (including shotgun data and closure results) indicate that the assembly is an accurate representation of the strain used for sequencing. Though it is possible the assembly is inaccurate, we believe it is more likely that there are genomic differences between the strain sequenced and strains used in our experiments. For instance, the H. volcanii DS2 strain was used to isolate DNA for sequencing (Table 3), whereas genetic and biochemical experiments used derivatives of H. volcanii WFD11 or DS70 [19,20].

**Table 3:**
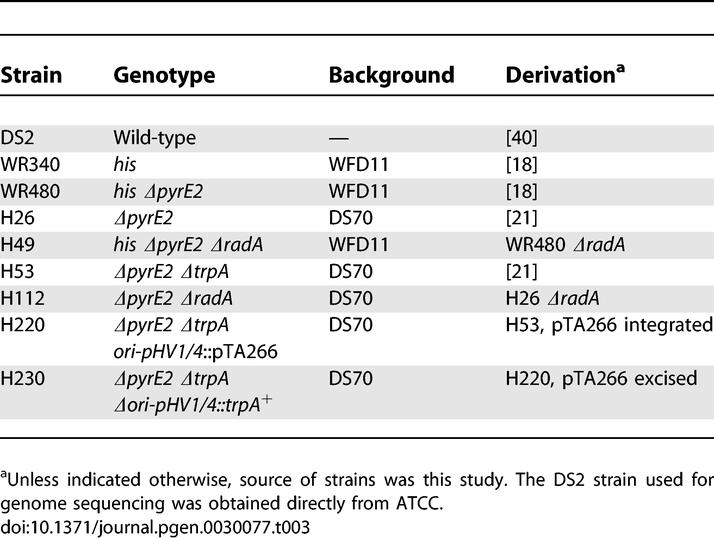
H. volcanii Strains

These discrepancies suggest that *H. volcanii,* whose genome is considered relatively stable compared to other halo-archaea, undergoes periodic genome arrangements that are possibly mediated by recombination at the replication origins. In this respect, it is noteworthy that the two rRNA operons are located close to the two chromosomal origins described in this work. The *rrnA* operon is 200 kb away from *oriC1,* and *rrnB* is only 6 kb away from *oriC2.* Homologous recombination between identical sequences at these *rrn* operons could provide a mechanism for origin movement throughout the genome. Intriguingly, the *rrn* operons are both oriented away from the origins, presumably to avoid collisions between replication fork and rRNA transcription machinery.

It is notable that the H. volcanii GC-skew plots do not delineate origins as clearly as the plots of the other sequenced haloarchaea (Figure 4). For example, the peak in contig 454 does not match the ARS isolated in the initial screen. Conversely, there is a peak in contig 455 that has higher amplitude than the two origins detected but is not associated with an experimentally determined origin (Figure 4A); this peak is most likely due to a recent integration by an AT-rich prophage. These quirks could indicate either that the H. volcanii “origin signal” (i.e., the skew caused by origin usage) is weak, or that the origin is mobile and has left behind a residual signal at its previous location. Finally, the fact that haloarchaeal genomes present “inverted” GC-skews indicates that the haloarchaea may not follow the same rules for genome structure patterning as in other species.

Given the apparent plasticity of the H. volcanii genome, we believe that pHV4, pHV3, and pHV1 should be considered as secondary chromosomes and not plasmids, in the sense that a plasmid is an extrachromosomal element and is often characterized by “selfish” behavior. Prokaryotic chromosome biology has been dominated by the archetype of *E. coli,* but there are many examples of bacteria with more than one chromosome [36]; like *H. volcanii,* they often show evidence of dynamic rearrangements between the replicons (e.g., [37]). The distinction between mini-chromosomes and mega-plasmids is usually made on the basis of replicon size and the presence of essential (housekeeping) genes. In this regard, both pHV4 and pHV3 are large (690 and 442 kb, respectively), and while genes predicted to be essential are only found on the former, H. volcanii strains that have lost pHV3 grow very slowly and filament [20]. More relevant to this work, pHV4, pHV3, and pHV1 all use Cdc6/Orc1-dependent origins for replication, as does the main chromosome. By contrast, pHV2 is very small (6.4 kb), lacks homology with chromosomal sequences, is easily cured, and uses a distinct (presumably Rep-dependent) replication origin [19]. Thus it is a plasmid.

In conclusion, we have shown that the four chromosomes of H. volcanii are replicated in an analogous manner using Cdc6/Orc1-dependent origins. Since efficient tools now exist to characterize the *cis*-acting requirements for replication initiation in archaea, H. volcanii will serve as an interesting model system for studies on the regulated replication of complex archaeal genomes.

## Materials and Methods

Unless stated otherwise, chemicals were from Sigma (http://www.sigma-aldrich.com) and enzymes from New England Biolabs (http://www.neb.com). Standard molecular techniques were used [38].

### Strains and plasmids.

H. volcanii strains are shown in Table 3, plasmids in Table 4, and oligonucleotides in Table S1.

**Table 4:**
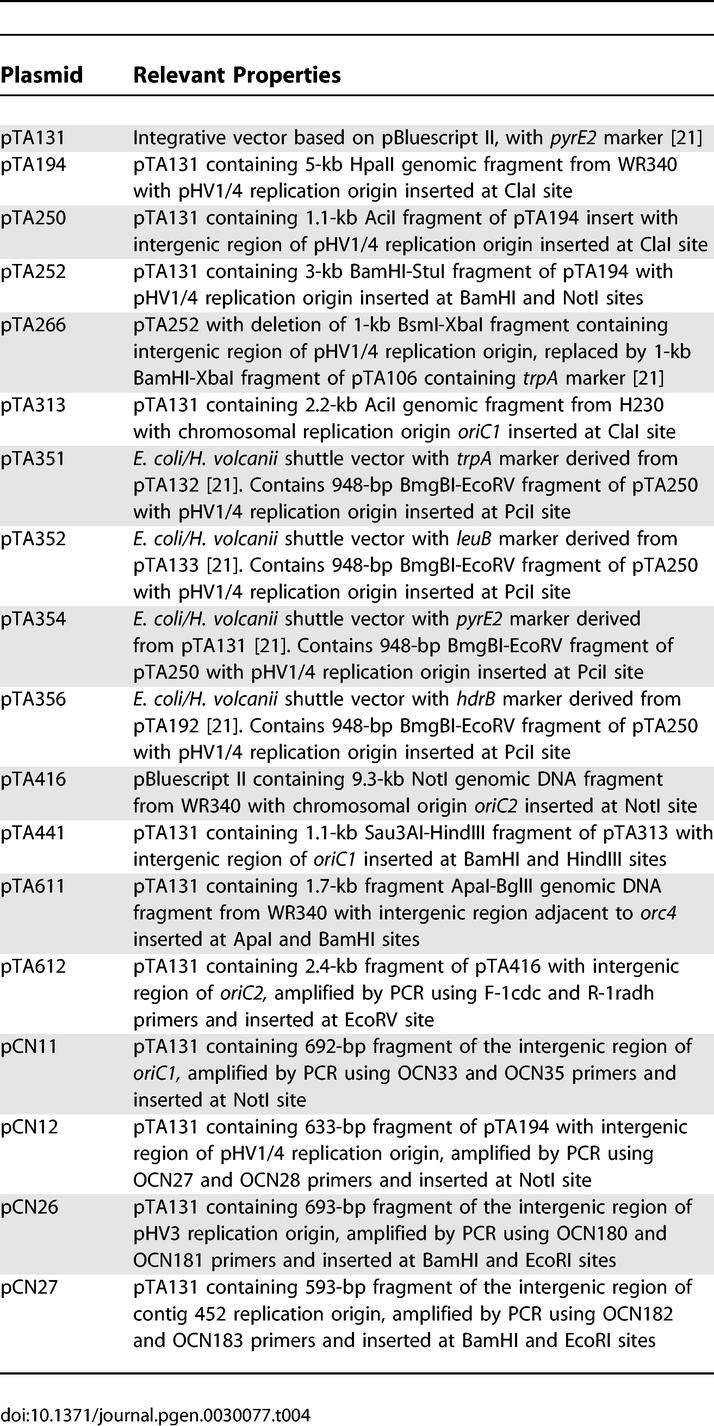
Plasmids

### Media and growth conditions.

H. volcanii strains were grown at 45 °C on either complete (Hv-YPC) or casamino acids (Hv-Ca) agar, or in Hv-YPC or casamino acids broth, as described previously [21,39].

### Molecular genetic methods.

Transformation of H. volcanii and isolation of total genomic DNA were carried out as described previously [21]. To isolate plasmid DNA, 2 ml of a saturated culture (grown in Hv-Ca broth) was centrifuged at 3,300 ×*g* for 8 min and the cells were resuspended in 50 μl of 1 M NaCl, 20 mM Tris HCl (pH 7.5); thereafter, a QIAprep miniprep kit (Qiagen, http://www.qiagen.com) was used according to the manufacturer's instructions. To isolate crude DNA, cells were resuspended in 400 μl of water and incubated at 70 °C for 10 min.

### Genomic DNA libraries.

H. volcanii DNA was digested with HpaII, AciI, or TaqI for 30 min at the recommended temperature, using ∼0.2 units of enzyme/μg DNA in suboptimal buffer (e.g., New England Biolabs buffer 1 for AciI). DNA fragments of the desired size were excised from agarose gels and ligated with plasmid pTA131 [21], which had previously been cut to completion with ClaI and the DNA ends dephosphorylated. The plasmid library was used to transform an *E. coli dam^–^* strain, and DNA was prepared directly from colonies to avoid differential amplification. Coverage of the libraries was >99.5% (>5,000 E. coli colonies were used in DNA preparation and H. volcanii has a genome size of ∼4 Mb). DNA was used to transform *H. volcanii pyrE2 radA* mutants H49 or H112, and transformants were selected on Hv-Ca plates lacking uracil.

### Deletion of pHV1/4 origin.

A 3-kb BamHI-StuI fragment of pTA194 with the pHV1/4 replication origin was subcloned to generate pTA252, and a 1-kb BsmI-XbaI fragment of the intergenic region was replaced by a *trpA* selectable marker [21] to generate pTA266 (Figure 2D). pTA266 was used to transform *ΔpyrE2 ΔtrpA* strain H53, and transformants were selected on Hv-Ca plates lacking uracil and tryptophan. One transformant (H220) was grown without selection for ∼30 generations and plated on Hv-Ca + 5-FOA (5-fluoroorotic acid) to select for loss of the integrated plasmid. Around 0.4% of cells in the culture were 5-FOA-resistant but only 0.13% of these were Trp^+^ and therefore had deleted the pHV1/4 replication origin.

### Estimation of ARS plasmid stability.

H112 containing pTA250 or pTA441 (maintained by selection on Hv-Ca agar) was used to inoculate a culture in Hv-YPC broth and grown at 45 °C. At regular intervals aliquots were plated Hv-YPC agar, and colonies patched on Hv-Ca to determine the fraction of uracil+ cells. % plasmid loss per generation *(l)* was calculated using the formula 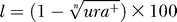 where *n* is the number of generations and *ura^+^* is the fraction of uracil+ cells.

### PFG electrophoresis.

Intact H. volcanii DNA was prepared in agarose plugs. 2 ml of culture (OD_650nm_ of 1.0) was pelleted at 3,300 ×*g* for 10 min, 4 °C, resuspended in 1 ml of cold spheroplasting solution (15% sucrose, 1 M NaCl, 27 mM KCl, 50 mM Tris-HCl [pH 8.5]) + 0.1% NaN_3_, and pelleted again. Cells were gently resuspended in 80 μl of spheroplasting solution, transferred to 42 °C, mixed with 100 μl of 1.5% low-melt agarose (in 0.5× spheroplasting solution, 100 mM EDTA) and pipetted into plug moulds (Bio-Rad, http://www.bio-rad.com). Plugs were incubated in 5 ml of lysis solution (1% sarkosyl, 500 mM EDTA, 20 mM Tris-HCl [pH 8.8]) + proteinase K (0.5 mg/ml) for 3 h at 52 °C, then transferred to fresh lysis buffer + proteinase K + RNaseA (30 mg/ml) and incubated overnight at 52 °C. Plugs were washed three times in 10 ml of 100 mM EDTA, 25 mM Tris-HCl (pH 7.5) at 37 °C, equilibrated in 0.5× Tris-borate-EDTA (TBE) for 90 min at 20 °C and exposed to 50 Gy of γ radiation (^137^Cs, 375 Gy/s) to linearize circular DNA molecules. Plugs were loaded onto a 1% agarose 0.5× TBE gel and electrophoresis was performed at 14 °C in a CHEF mapper (Bio-Rad) using 0.5× TBE buffer, voltage gradient of 6 V/cm, switch angle of 120°, and switch times of 0.47 s (initial) to 1 min 33.83 s (final). Total run time was 20 h 18 min.

### RIP mapping assay.

H. volcanii DS2 was diluted to an OD_650nm_ of 0.15 in 100 ml of Hv-YPC media, grown to OD_650 nm_ of 0.3, and pelleted. Cells were resuspended in 4 ml of lysis buffer (25 mM Tris-HCl [pH 7.5], 20 mM EDTA, 100 mM NaCl, 200 μg ml^−1^ proteinase K, 1% SDS). After 1 h incubation at 50 °C, 4 g of CsCl and 100 μl of Hoechst-33342 (5 mg ml^−1^) were added and the refractive index adjusted to 1.410 with CsCl (1 g ml^−1^). DNA was purified by CsCl gradient ultracentrifugation. To enrich for replicating intermediates, total DNA was passed down a BND-cellulose column pre-equilibrated with NET buffer (10 mM Tris-HCl [pH 8.0], 1 mM EDTA, and 1 M NaCl) to selectively bind single-stranded DNA. After washing with NET buffer, bound DNA was eluted with NET buffer + 1.8% caffeine at 50 °C. DNA was isopropanol-precipitated and resuspended in TE buffer (10 mM Tris-HCl [pH 7.5], 1 mM EDTA) at 1 μg μl^−1^. After phosphorylation of 5′-OH ends with T4 polynucleotide kinase (Promega, http://www.promega.com), DNA was treated with λ-exonuclease to digest 5′ nicked DNA ends; replication intermediates protected by RNA primers are unaffected by this treatment. Primer extension reactions used ∼500 ng of enriched replicating intermediates, 25 ng of radiolabeled primer (labeled using [γ^32^P]-ATP, and T4 polynucleotide kinase) and 2 units of Deep Vent (exo-) DNA polymerase. After 30 cycles of reaction (60 s at 94 °C, 60 s at 70 °C, and 90 s at 72 °C), amplification products were separated on a 6% polyacrylamide gel under denaturing conditions. Radioactive material was detected using a Phosphorimager system (Amersham, http://www.gehealthcare.com). Control experiments using linearized plasmid DNA isolated from E. coli were performed under similar conditions.

### Bioinformatics.

Nucleotide representation disparities were calculated using either ORIGINX (http://www.cbs.dtu.dk/services/GenomeAtlas/suppl/origin) or ZPLOTTER (http://tubic.tju.edu.cn/zcurve) programs. Publicly available sequences released in June 2006 were used to calculate local minima and maxima in nucleotide disparities. DNA curvature was calculated using a BEND.IT server (http://hydra.icgeb.trieste.it/∼kristian/dna/bend_it.html), which predicts in qualitative terms a curvature propensity of a given DNA sequence using DNase I based bendability parameters [41] and the consensus bendability scale [42]. The resulting data of each program were plotted using Origin Pro 7.5 software (OriginLab Corporation, http://www.originlab.com).

## Supporting Information

Figure S1 *E. coli/H. volcanii* Shuttle Vectors with *ori-pHV1/4*
Shuttle vectors pTA351, pTA352, pTA354, and pTA356 are derived from pTA132, pTA133, pTA131, and pTA192, respectively [21], and feature the DNA replication origin from pHV1/4 isolated in the initial genetic screen.
(470 KB PDF)

Figure S2 Colony Size of H. volcanii Transformed with ARS Plasmids H. volcanii H112 *(ΔpyrE2 ΔradA)* was transformed with pTA250 *(ori-pHV1/4),* pTA441 *(oriC1),* pTA612 *(oriC2),* pCN26 *(ori-pHV3),* or pCN27 *(ori-pHV4–2).* Cells were diluted 100-fold and 100 μl was plated on Hv-Ca, except for pCN27 where transformants were plated without dilution. Plates were incubated at 45 °C for 15 d before photographs were taken.
(3.3 MB PDF)

Table S1 Oligonucleotides (52 KB DOC)

## Abbreviations

5-FOA: 5-fluoroorotic acid
ARS: autonomously replicating sequence
DUE: duplex unwinding element
MCM: minichromosome maintenance
ORB: origin recognition box
ORC: origin recognition complex
PFG: pulsed field gel
RIP: replication initiation point
