# Supplementary material for: Genetic and Physical Mapping of DNA Replication Origins in Haloferax volcanii
Source: PLoS Genet. 2007 May 18;3(5):e77. doi: 10.1371/journal.pgen.0030077 (PMC1868953; doi:10.1371/journal.pgen.0030077)
Supplement: Supplementary file 2 [file pgen.0030077.sg002.pdf]

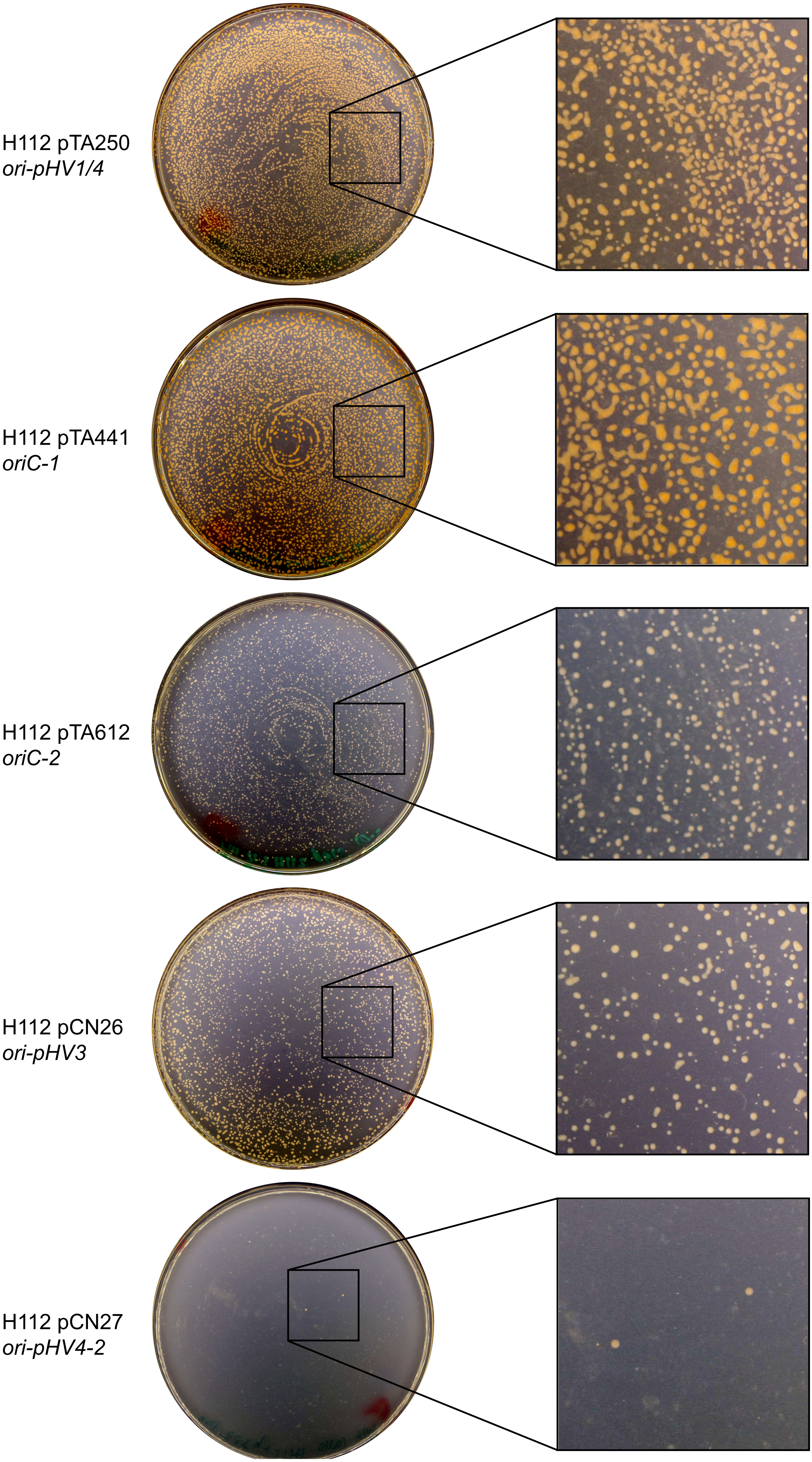

Supplemental Figure 2
