# Supplementary material for: Genetic and Physical Mapping of DNA Replication Origins in Haloferax volcanii
Source: PLoS Genet. 2007 May 18;3(5):e77. doi: 10.1371/journal.pgen.0030077 (PMC1868953; doi:10.1371/journal.pgen.0030077)
Supplement: Supplementary file 3 [file pgen.0030077.st001.doc]

**Supplemental Table 1.** Oligonucleotides

| **Primer** | **Sequence (5'–3')** | **Relevant properties** |
| --- | --- | --- |
| F-1cdc | TCCCAGTTTTCCCGTAGATGAGG | Forward primer for amplification of intergenic region upstream of *orc5* |
| R-1radh | TCGGATACCTCGGTTACGTCG | Reverse primer for amplification of intergenic region upstream of *orc5* |
| OCN24  RIP-e | AGAGGAACTCATCGACTGCGTGGAG | Forward primer for RIP mapping of *ori-pHV1/4* |
| OCN27 | ACACCCTCATGTCGAGTGTGTCGAA | Forward primer for amplification of intergenic region upstream of *orc10* |
| OCN28 | GGTCGGTAGAACGGTGCTGTTGACT | Reverse primer for amplification of intergenic region upstream of *orc10* |
| OCN29  RIP-f | TCGCAAGACAGTGTTGGAATCACG | Reverse primer for RIP mapping of *ori-pHV1/4* |
| OCN33  RIP-a | CGTAACTCGAACGGATACCGAGCAT | Forward primer for RIP mapping of chromosomal replication origin *oriC1* |
| OCN34  RIP-b | CGAATAACGATTTGGACGAATTCGG | Reverse primer for RIP mapping of chromosomal replication origin *oriC1* |
| OCN35 | GTGACTGAACGATGGTGGGTAGCC | Reverse primer for amplification of intergenic region upstream of *orc1* |
| OCN180 | cgggatccGTGGTTGGGAGGTTGTGAAC | Forward primer for amplification of intergenic region upstream of *orc6* |
| OCN181 | ggaattcTGGACTCGCGTTTGTAACAG | Reverse primer for amplification of intergenic region upstream of *orc6* |
| OCN182 | cgggatccATGTCCGCAGTTGTTCGTC | Forward primer for amplification of intergenic region upstream of *orc3* |
| OCN183 | ggaattcACTTAGGCGGGAACACACC | Reverse primer for amplification of intergenic region upstream of *orc3* |
| OCN254  RIP-c | CGATGAGTCACGAGAGCGAGGTCAC | Forward primer for RIP mapping of chromosomal replication origin *oriC2* |
| OCN126  RIP-d | CCGGTGAAATACGATACAGGGCAGAC | Reverse primer for RIP mapping of chromosomal replication origin *oriC2* |
| OCN240  RIP-g | TGGTTGGGAGGTTGTGAACTGGAA | Forward primer for RIP mapping of replication origin *ori-pHV3* |
| OCN241  RIP-h | GTGTGTGTTCCCGAGGACAGATGG | Reverse primer for RIP mapping of replication origin *ori-pHV3* |
| OCN244  RIP-i | GGTCGAGACGTGTTCGGTGAGTGT | Forward primer for RIP mapping of replication origin *ori-pHV4-2* |
| OCN246  RIP-j | GGAACACACCCCCTCCACTTTGTC | Reverse primer for RIP mapping of replication origin *ori-pHV4-2* |
